# Stereoscopic X-ray imaging, cone beam CT, and couch positioning in stereotactic radiotherapy of intracranial tumors: preliminary results from a cross-modality pilot installation

**DOI:** 10.1186/s13014-016-0735-2

**Published:** 2016-12-07

**Authors:** Barbara Zollner, Christian Heinz, Sabrina Pitzler, Farkhad Manapov, Steffi Kantz, Maya Christine Rottler, Maximilian Niyazi, Ute Ganswindt, Claus Belka, Hendrik Ballhausen

**Affiliations:** LMU Munich, Department of Radiation Oncology, Marchioninistraße 15, Munich, 81377 Germany

**Keywords:** Image guided radiotherapy, Stereotactic radiotherapy, Radiosurgery, Stereoscopic X-ray imaging, Cone beam computed tomography, Patient positioning, Intracranial tumors

## Abstract

**Background:**

To assess the accuracy and precision of a fully integrated pilot installation of stereoscopic X-ray imaging and kV-CBCT for automatic couch positioning in stereotactic radiotherapy of intracranial tumors. Positioning errors as detected by stereoscopic X-ray imaging are compared to those by kV-CBCT (i.e. the accuracy of the new method is verified by the established method), and repeated X-ray images are compared (i.e. the precision of new method is determined intra-modally).

**Methods:**

Preliminary results are reported from a study with 32 patients with intracranial tumors. Patients were treated with stereotactic radiotherapy guided by stereoscopic X-ray imaging and kV-CBCT. Patient positioning was automatically corrected by a robotic couch. Cross-modal discrepancies in position detection were measured (*N =* 42). Intra-modal improvements after correction and re-verification by stereoscopic X-ray imaging were measured (*N =* 70). The accuracy and precision of stereoscopic X-ray imaging and the accuracy and precision of CBCT were confirmed in phantom measurements (*N =* 12 shifts of a ball bearing phantom, *N =* 24 shifts of a head phantom).

**Results:**

After correction based on stereoscopic X-ray imaging 95% of residual mean errors were below 0.4, 0.4, 0.5, and 0.7 mm (lateral, longitudinal, vertical, radial, respectively). Stereoscopic X-ray imaging and CBCT were in close agreement with an average discrepancy of 0.1, 0.5, 0.3 and 0.8 mm, respectively. 95% of discrepancies were below 0.8, 1.2, 1.0, and 1.4 mm, respectively. After correction and re-verification by stereoscopic X-ray imaging, the remaining intra-modal residual error was consistent with zero (*p =* 0.31, *p =* 0.48, *p =* 0.81 in lateral, longitudinal, and vertical direction; *p-*values from two-tailed *t*-test). The inherent technical accuracy and precision of stereoscopic X-ray imaging and the accuracy and precision of CBCT were found to be of the order of 0.1 mm in controlled phantom settings.

**Conclusions:**

In a routine clinical setting, both stereoscopic X-ray imaging and CBCT were able to reduce positioning errors by an order of magnitude. The end-to-end precision of the system, measured from the discrepancy (mean) between ExacTrac and CBCT, in a clinical setting seems to be about 0.8 mm radially, including couch positioning. The precision (measured from repeatability of ExacTrac, intra-modal) was found to be about 0.7 mm radially in a clinical setting.

## Background

Frameless linac-based image-guided radiosurgery has a significant role in the treatment of brain tumors and metastases [1–5]. Both single-fraction and (hypo)fractionated stereotactic radiotherapy require a precise positioning of the patient and target alignment [6].

There are different image-guidance systems available, including kilo-voltage (kV) or megavoltage (MV) X-ray-imaging, kV or MV cone-beam CT (CBCT) or MV single slice CT (tomotherapy) [7, 8]. Most commonly used are kV X-ray imaging and CBCT [8]. The integration of more than one imaging modality has improved the management of geometric uncertainties in contemporary radiotherapy practice. Some of the image-guidance systems also allow monitoring intra-fraction organ movement [9]. In addition, there are radiation free methods such as surface imaging (e.g. AlignRT [10]), surface laser scanning (e.g. Sentinel [11]) and time-of-flight cameras (e.g. [12, 13]).

At our institution a hybrid system was installed, using kV stereoscopic X-ray imaging (ExacTrac, Brainlab AG, Feldkirchen, Germany) and kV-CBCT (on-board imager from Versa HD, Elekta AB, Stockholm, Sweden). The ExacTrac system offers translational and rotational parameters for stereotactic patient positioning [8]. All six degrees of freedom can be verified and corrected at any time before or during treatment [6]. For the provision of the planar images, less radiation dose to the patient is required than during a CBCT [8]. In addition, planar images may also be acquired at non-zero couch angles where CBCT acquisition is not possible due to the risk of collision of the gantry with the treatment couch. In certain circumstances, kV-CBCT may provide a better visualization of anatomical structures and soft tissues on transaxial images [8, 14, 15]. Combining both systems may result in superior positioning accuracy and/or precision.

In this study we evaluated the 3 DOF positioning accuracy and precision and compared the residual setup errors measured with ExacTrac and kV-CBCT. Both a cross-modality comparison (to judge comparability) and an intra-modality verification (to judge repeatability) are presented. Specifically, positing errors as detected by stereoscopic X-ray imaging are compared to those by kV-CBCT (i.e. the accuracy of the new method is verified by the established method), and repeated x-ray images are compared (i.e. the precision of new method is determined intra-modally).

Also, the improvement of residual positioning errors before and after verification and automatic table correction was evaluated.

In addition to the retrospective evaluation of patient data from routine clinical workflow, the inherent technical limit of both ExacTrac and CBCT were tested in controlled phantom experiments.

## Methods

**Fig. 1:**
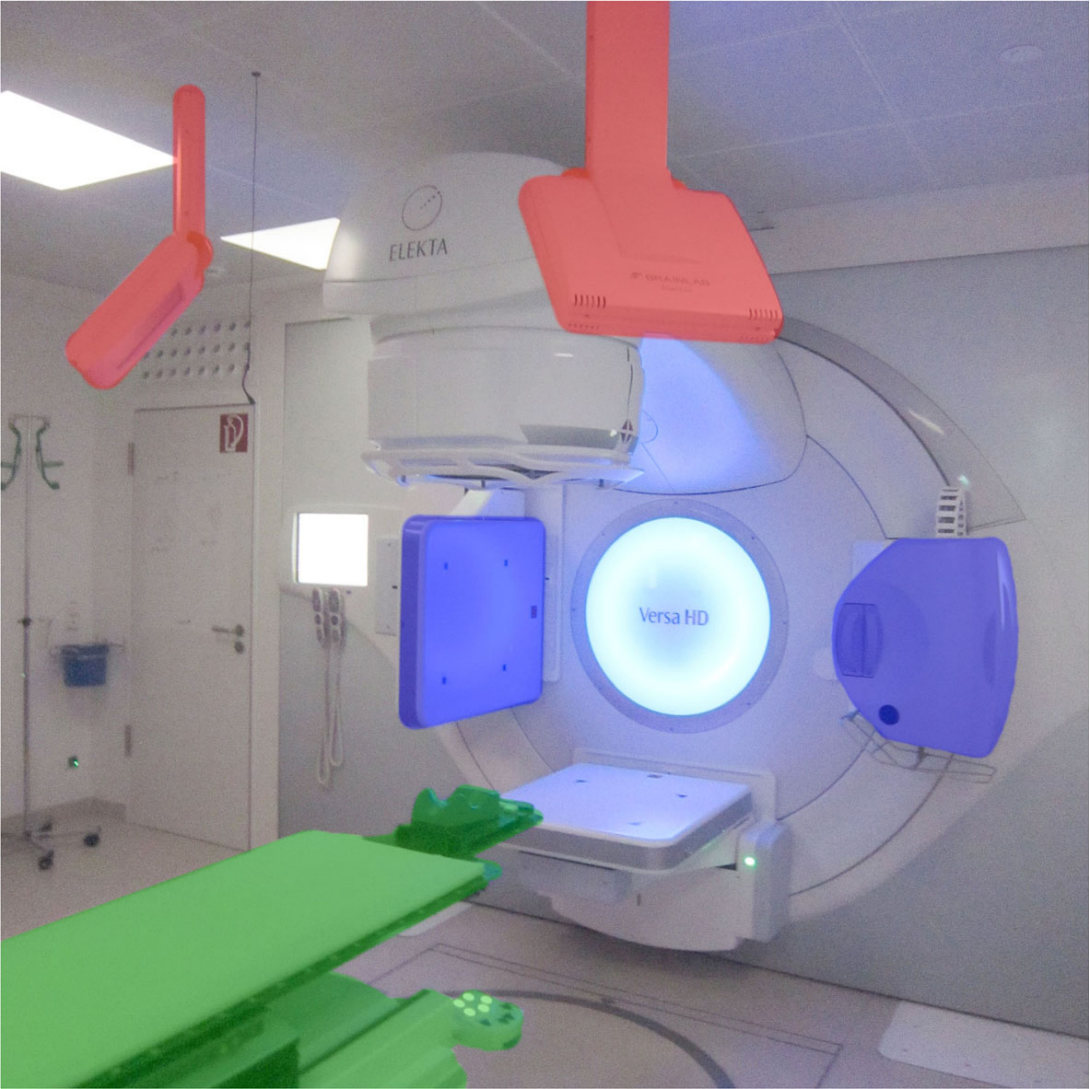
Installation of Elekta Versa HD with integrated kV-CBCT (*highlighted in blue*), Brainlab ExacTrac stereoscopic X-ray imaging (detectors highlighted in red, sources are hidden in floor) and robotic patient couch with 6 DOF Hexapod (*highlighted in green*)

In collaboration with Elekta AB, Stockholm, Sweden, and Brainlab AG, Feldkirchen, Germany, a pilot installation has been set up for fully integrated IGRT of intracranial tumors at our institution, see Fig. 1. The installation was comprised of 1. Elekta Versa HD linear accelerator with integrated CBCT, 2. stereoscopic X-ray imaging of type ExacTrac by Brainlab, 3. either a Brainlab mask or an iCAST Head Micro Double-mask system, 4. frame fixed to the patient table, with infrared markers for detection by a pair of stereoscopic cameras, and 5. hexapod patient couch for automatic correction. The installation was particular in that it was one of the first two installations to offer seamless end-to-end integration of the three modalities (ExacTrac, CBCT, and IR markers) with the robotic couch. The installation provides six degrees of freedom, and both translations and rotations (6 DOF via Hexapod) are used clinically. This study is only concerned with translational degrees of freedom and reports values for three axes and radial (3D) vector lengths.

For the CBCT imaging system, a preset by the manufacturer was used. This ‘fast head and neck S20’ setting entails 100 kV tube voltage with 10 mA current and 10 ms exposure time per frame for a total of 18.3 mAs in 183 frames over a gantry rotation angle span of 205° from −135° to +70°. Acquisition parameters of the ExacTrac x-ray imaging system were 80 kV times 6.30 mAs (standard settings for cranial imaging). Rigid registration and automatic fusion based on bony features was performed by the proprietary matching algorithm of the instrument software, without manual intervention.

### Patients

Between February 2015 and July 2015, 32 patients with intracranial tumors underwent stereotactic radiotherapy at the installation. Planning CTs were acquired at a separate site with identical mask setup and 1 mm slice thickness. For treatment, patients were put on the robotic couch and were fixated with stereotactic masks.

#### Cross-modality comparison of CBCT, and ExacTrac

The couch was first roughly pre-positioned by hand, then automatically adjusted via the infrared markers. The position was verified by ExacTrac and/or CBCT. Discrepancies between ExacTrac and CBCT were analyzed and the accuracy and precision of the two modalities was compared.

#### Intra-modality improvement after correction and re-verification by ExacTrac

The position of the couch was corrected when prompted by ExacTrac and fine-positioned based on ExacTrac readings. About half of the corrected positions were re-verified by ExacTrac. Positioning errors before and after correction were analyzed.

### Intra-modality repeatability of position detection by ExacTrac

Finally, in some cases when a discrepancy seemed larger than expected, ExacTrac was immediately repeated without a correction to verify the result. These measurements were used to analyze the repeatability of ExacTrac.

### Phantoms

#### Ball bearing phantom

In a separate phantom measurement, a fixed ball bearing phantom was used. This phantom (type Elekta Synergy Basic Calibration Kit MRT 15991) consisted of a rod of acrylic glass of about 30 cm of length and about 1 cm in diameter with an enclosed steel ball bearing of 8 mm in diameter at the far end. The rod was fixed to a set of three mounts adjustable by micrometer screws for three translational degrees of freedom. The position of the phantom was determined repeatedly (*N =* 10) by ExacTrac. The standard deviation of these position readings was used as a measure of repeatability. Then, the phantom was shifted by ±1 mm in either direction (twice per axis, for a total of *N =* 12 shifts detected by 24 independent position readings) by a micrometer screw with a precision of 0.5 mm travel per turn/50 marks per tur*N =* 0.01 mm. The average ± SD deviation of the detected shift from 1 mm was calculated as a measure of absolute accuracy and precision of shift detection.

#### Head phantom

In a second phantom measurement, a head phantom (type SK 150 by manufacturer The Phantom Laboratory, Incorporated, P.O. Box 511, Salem NY 12865, USA; phantomlab.com; real human skull moulded in tissue equivalent resin; internal air cavities represent the oral, pharynx and trachea anatomy’) was repeatedly moved by small amounts by the robotic couch. Per axis, shifts of ± 5 mm and ± 10 mm were repeated twice each, for a total of *N =* 24 shifts (comprised of 48 statistically independent position readings). The maximum shift of 10 mm was motivated by clinical data as the largest shift ever needed in this set of patients after pre-positioning was 7.7 mm laterally. The shifts were detected both by ExacTrac and by CBCT and differences were compared.

For the *N =* 24 scans several times during the workflow were recorded. Focus was on the technical durations of key steps, with user interaction excluded as far as possible: the time necessary to turn the gantry to the 180° starting position for the CBCT scan (only the rotation time from start to stop), the time necessary to turn the gantry to the off-angle position needed for ExacTrac (only the rotation time from start to stop), the duration of the 360° CBCT scan (only the scanning time from pressing the release button to the completion of the scan), the duration of the ExacTrac exposure (from pressing the release button to the appearance of the image on the screen), the duration of the automatic CBCT registration (only the iteration computation time, without any previous user interaction, from pressing the registration button to the termination of the iteration), and the duration of the automatic ExacTrac registration (only the iteration computation time, without any previous user interaction, from pressing the registration button to the termination of the iteration). While these times were not comparable to clinical workflow times and neglect e.g. patient positioning, user interaction, checking registration results, bookkeeping etc. they were more representative of the purely technical characteristics of the two modalities.

Statistical analysis was performed with Microsoft Excel, Version 2010. All *p-*values were calculated by the two-tailed *t*-test.

## Results

### Patient characteristics and treatment parameters

Thirty-two patients were included in the analysis. 18 (56%) were female, and 14 (44%) were male. Median age at treatment was 66.8 years, range 40.0 to 83.3 years. All patients had developed brain metastases, mainly from lung cancer (16 cases), malignant melanoma of the skin (7 cases), breast cancer (2 cases), and miscellaneous (7 cases).

In total, 46 target volumes had been defined. 37 of them required a single dose of 20 Gy at 80% (22 cases), 18 Gy at 80% (12 cases), or 15 Gy at 80% (3 cases). The other 9 target volumes were treated with 5 fractions of each 5 Gy at 80% [16]. Hence, in total 37 × 1 + 9 × 5 = 82 fractions were delivered, with typically 4 to 6 fields for different couch angles per fraction, and all fields and fractions were all included in this analysis.

#### Cross-modality comparison of CBCT and ExacTrac

In 42 out of the 82 fractions, both a CBCT and an ExacTrac verification were available after positioning on infrared markers at 0° couch position. On average, the detection by CBCT and ExacTrac differed by +0.1 ± 0.3 mm in the lateral direction (median +0.1 mm, range −0.9 to +0.8 mm), by −0.5 ± 0.4 mm in the longitudinal direction (median −0.4 mm, range −1.6 to +0.2 mm), by −0.3 ± 0.5 mm in the vertical direction (median −0.4 mm, range −1.4 to +0.8 mm), and by 0.8 ± 0.3 mm (median 0.8 mm, range 0.3 to 1.8 mm) radially (i.e. Euclidean 3D distance). 95% of the discrepancies were below 1.4 mm radially at most. See Table 1.

**Table 1:** Discrepancies in position detection between ExacTrac and CBCT (*N =* 42)

	mean ± SD	median	range	95% (abs.)
lateral	+0.1 ± 0.3	+0.1	–0.9 to +0.8	<0.8
longitudinal	–0.5 ± 0.4	–0.4	–1.6 to +0.2	<1.2
vertical	–0.3 ± 0.5	–0.4	–1.4 to +0.8	<1.0
radial	0.8 ± 0.3	0.8	0.3 to 1.8	<1.4

*CBCT* Cone Beam Computed Tomography

All units are in mm

Very similar results were found in the subgroup using the Brainlab mask (*N =* 15) and the subgroup using the iCAST double mask. The respective discrepancies between CBCT and ExacTrac were 0.0 ± 0.2 mm vs. 0.1 ± 0.4 mm in the lateral direction (*p =* 0.13), −0.5 ± 0.3 mm vs. -0.4 ± 0.4 mm in the longitudinal direction (*p =* 0.32), and −0.1 ± 0.5 mm vs. -0.4 ± 0.5 mm in the vertical direction (*p =* 0.08). Judging from these *p-*values, no significant influence of the mask system could be identified.

#### Intra-modality improvement after correction and re-verification by ExacTrac

One hundred thirteen times, the positioning error detected by the ExacTrac ‘verification’ (i.e. measurement of the initial error after positioning on IR markers only) required a correction of the couch position, each followed by re-verification (i.e. measurement of the remaining error after positioning based on the former ExacTrac verification). Out of the 113 times, 43 times a rotation of the couch was required, but 70 times the re-verification was performed at the same couch angle. Only these 70 values entered the following calculation. Before correction, the average error was of the order of one millimeter per axis. After correction, the average residual error was lower by one order of magnitude, and the maximum residual error was of the order of one millimeter, see Table 2. In particular, 95% of radial errors were below 6.1 mm before correction and below 0.69 mm after correction.

**Table 2:** Improvement after correction and re-verification by ExacTrac (*N =* 70)

	mean ± SD	median	range	95% (abs.)
Before correction by ExacTrac				
lateral	–0.7 ± 2.3	–0.7	–7.7 to +5.9	<5.2
longitudinal	+0.3 ± 1.5	+0.2	–3.6 to +4.6	<3.5
vertical	–1.2 ± 1.8	–0.8	–10.7 to +1.3	<3.1
radial	2.9 ± 2.0	2.3	0.2 to 11.8	<6.1
After correction by ExacTrac				
lateral	+0.03 ± 0.25	+0.02	–0.40 to +1.13	<0.38
longitudinal	–0.02 ± 0.21	–0.04	–0.54 to +0.95	<0.40
vertical	+0.01 ± 0.22	+0.01	–0.67 to +0.56	<0.51
radial	0.32 ± 0.22	0.26	0.07 to 1.33	<0.69

All units are in mm

The remaining residual error was consistent with zero (*p =* 0.31, *p =* 0.48, *p =* 0.81 by the two-tailed *t*-test in lateral, longitudinal, and vertical direction).

#### Intra-modality repeatability of position detection by ExacTrac

Fourteen times a measurement by ExacTrac was immediately repeated without a correction, in case the discrepancy seemed larger than expected, to verify the result. Repeatability was within 1 mm at least and typically around half a millimeter, see Table 3. In particular, radial repeatability was below 0.7 mm in 50% of cases and below 1.2 mm in 95% of cases.

**Table 3:** Repeatability of position detection by ExacTrac (*N =* 14)

	mean ± SD	range	95% (abs.)	50% (abs.)
Difference before – after				
lateral	–0.08 ± 0.52	–0.94 to +0.53	<0.8	<0.5
longitudinal	–0.05 ± 0.70	–0.74 to +1.01	<0.8	<0.1
vertical	–0.03 ± 0.42	–0.67 to +0.83	<0.7	<0.3
radial	0.73 ± 0.32	0.26 to 1.28	<1.2	<0.7

All units are in mm

### Confirmation of absolute accuracy and precision in a phantom measurement

#### Ball bearing phantom

Of 10 repeated measurements of the positon of a fixed ball bearing phantom, the standard deviation of measurements by ExacTrac was smaller than 0.03 mm in either direction.

Of 12 shifts of the ball bearing phantom of exactly 1 mm ± 0.001 mm, all shifts detected by ExacTrac were within 0.1 mm of the true shift. The average ± SD discrepancy was −0.01 ± 0.04 mm in vertical direction, 0.01 ± 0.01 mm in longitudinal direction, and −0.02 ± 0.10 mm in lateral direction.

#### Head phantom

The ‘reference’ shifts of the robotic couch and the head phantom were quite precisely detected by both ExacTrac and CBCT. Both modalities showed non-significant discrepancies with standard deviations between 0.3 and 0.6 mm. However, the discrepancy between ExacTrac and CBCT was smaller still, of the order of 0.0 ± 0.1 mm. See Table 4. Most probably, both ExacTrac and CBCT are more exact than the patient couch itself and the limiting factor was in the ‘reference’ shift. Also, there were no detectable differences > 0.1 mm on either of the off-axes in either comparison. In particular, radial absolute differences were below 0.7 mm in 95% of cases.

**Table 4:** Shifts of a head phantom as preset by the robotic couch and as detected by both ExacTrac and CBCT (*N =* 24, resp. 8 per axis)

Discrepancy between …	… ExacTrac and robotic couch	… CBCT and robotic couch	… ExacTrac and CBCT
lateral	0.0 ± 0.5 (*p =* 0.9)	0.0 ± 0.6 (*p =* 1.0)	0.0 ± 0.1 (*p =* 0.5)
longitudinal	0.4 ± 0.3 (*p =* 0.1)	0.3 ± 0.3 (*p =* 0.1)	0.1 ± 0.1 (*p =* 0.3)
vertical	0.0 ± 0.4 (*p =* 0.9)	0.0 ± 0.4 (*p =* 1.0)	0.0 ± 0.1 (*p =* 0.5)
radial	0.7 ± 0.3 (*p =* 0.007)	0.7 ± 0.3 (*p =* 0.006)	0.1 ± 0.0 (*p <* 0.001)

All units are in mm, average ± SD (*p* values from two-sided *t*-test)

As regards the time necessary to perform a scan and registration, the following times were recorded during the head phantom experiment, see Table 5.

**Table 5:** Time requirements during the workflow of the head phantom measurement (*N =* 24)

	Gantry in position	Scan	Registration
ExacTrac	15.7 ± 2.9	6,8 ± 0.3	4.1 ± 0.2
CBCT	25.1 ± 1.1	41.2 ± 0.2	8.2 ± 2.0

All units are in seconds, average ± SD

Both ExacTrac and CBCT required the gantry in a suitable position before the scan. However, the required time will depend in practice on field configurations and thus the above values are only indicative of the order of magnitude and may not be representative of individual cases. The largest difference in relative and absolute terms is the scanning time, where ExacTrac is substantially faster, mainly because no further rotation of the gantry is necessary. The scan time registered includes the full workflow time with screen interaction, X-ray release, and screen update. The very X-ray exposure is much shorter. The times given for registration also include screen interaction time (around 1 s). The CBCT algorithm converges step-wise to the solution and thus runs the longer the larger the detected shift is. Without shift, 5 s are sufficient; with 10 mm shift, 10 s are needed; this explains the comparatively large standard deviation (2 s), whereas the ExacTrac registration time is independent of the shift (SD of 0.2 s).

## Discussion

This study compared measurements by ExacTrac and CBCT. The two modalities were found to be in close agreement on a scale of about one millimeter.

In a more controlled phantom setting, both ExacTrac and CBCT agreed to about 0.1 mm in precision; the limitation of the system is here in the accuracy of the robotic couch corrections.

Our study is most similar to Ma et al. [8] who report discrepancies between ExacTrac and (online 3D-) CBCT both for a phantom and patients. They report a translator root-mean-square error RMS ± SD of 0.26 ± 0.25 mm laterally, 0.34 ± 0.34 mm longitudinally, and 0.39 ± 0.38 mm vertically for a phantom, resp. 0.65 ± 0.63 mm laterally, 0.88 ± 0.82 mm longitudinally, and 1.23 ± 1.14 mm vertically for patients. This is in good agreement with our findings: expressed in RMS, we find 0.34 ± 0.42 mm laterally, 0.60 ± 0.71 mm longitudinally, and 0.58 ± 0.61 mm vertically for patients which is just in between their patient and phantom results.

Kim et al. [17] report a measurement of the accuracy (mean) and precision (SD) of ExacTrac in comparison to a reference CBCT of a pelvis phantom as −0.2 ± 0.2 mm laterally, −0.8 ± 0.4 mm longitudinally,–0.8 ± 0.2 mm vertically, and 1.2 ± 0.3 mm radially (3D vector length). This is in good agreement with our finding of −0.1 ± 0.3 mm laterally, −0.5 ± 0.4 mm longitudinally, −0.3 ± 0.5 mm vertically and 0.8 ± 0.3 mm radially (3D vector length). The slight differences in accuracy (mean) are most probably due to individual instrument calibration. As no N is given by Kim et al., no p value can be calculated about the significance of their and our finding. The higher precision (lower SD) in their measurement is probably due to the fact that they were using a phantom, while our figures were acquired from patients during routine clinical workflow conditions.

Similarly, Tominaga et al. [18] report a positioning error of ExacTrac in comparison to CBCT for a cubic phantom with enclosed spherical targets. At the isocenter, in the absence of rotations, they find a radial (3D vector length) accuracy (mean) and precision (SD) of 0.5 ± 0.1 mm. Again, no N is given, but their result does not seem to significantly differ from ours. In particular, the precision of 0.1 mm is the same as the precision in our phantom data.

The manufacturer Brainlab cites an accuracy of 0.68 and a precision of about 0.8 mm for ‘frameless radiosurgery (=ExacTrac) for a phantom’ in an ‘end-to-end’ test in their white paper [19, 20]. This study in patients took intra-fraction motion into account and was based on intra-modal comparisons and is thus difficult to compare to the present study.

Infusino et al. [21] as well as Oh et al. [22] report inter-fractional daily setup errors measured with ExacTrac with a focus on margin calculation rather than on the accuracy and precision of the system itself. Wurm et al. [23] similarly present intra-modal data and find ‘an overall system accuracy of 1.04 ± 0.47 mm’ (for a phantom) and an ‘overall average setup error of 0.31 ± 0.26 mm for translation’ (in patients). Finally, Keeling et al. use statistical modeling to separate uncertainties from mask, localizer, IR-frame, X-ray imaging, MV, and kV isocentry. They report a standard deviation (precision) of the X-ray imaging system of 0.16 mm radially, which again is in good agreement with our results.

As far as recommendations are concerned, the achieved accuracy is of the order of the geometric uncertainties mentioned by DIN 6875–1:2004–01 (0.5 to 2 mm) and well within the limits listed in the report no. 54 of AAPM Task Group 42 on stereotactic radiosurgery, compare page 7, table II [24].

## Conclusions

Automatic couch correction after verification was able to reduce patient positioning errors by an order of magnitude. The precision of the system, measured from the discrepancy (mean) between ExacTrac and CBCT, in a clinical setting seems to be about 0.8 mm radially, including couch positioning. Precision (measured from repeatability of ExacTrac, intra-modal) was found to be about 0.7 mm in a clinical setting. Inherent technical limitations to the precision of either modality in controlled phantom settings, by comparison, are only of the order of 0.1 mm.

## Abbreviations

CBCT: Cone beam computed tomography
CT: Computed tomography
SD: Standard deviation
